# A Novel Rotablator Technique (Low-Speed following High-Speed Rotational Atherectomy) Can Achieve Larger Lumen Gain: Evaluation Using Optimal Frequency Domain Imaging

**DOI:** 10.1155/2019/9282876

**Published:** 2019-05-20

**Authors:** Takanobu Yamamoto, Sawako Yada, Yuji Matsuda, Hirofumi Otani, Shunji Yoshikawa, Taro Sasaoka, Yu Hatano, Tomoyuki Umemoto, Daisuke Ueshima, Yasuhiro Maejima, Kenzo Hirao, Takashi Ashikaga

**Affiliations:** The Department of Cardiovascular Medicine, Tokyo Medical and Dental University, Japan

## Abstract

**Background:**

While the evaluation of burr speed was discussed regarding platelet aggregation, the association between platform speed and acute lumen gain of rotational atherectomy remains unknown.

**Methods:**

Through the evaluation of the potential of low-speed rotational atherectomy (LSRA) in* in-vitro* experiments, minimum lumen diameter (MLD) and minimum lumen area (MLA) after conventional high-speed rotational atherectomy (HSRA group) and those after LSRA following HSRA (LSRA+HSRA group) treated by 1.5 mm burrs were measured by optical frequency domain imaging (OFDI) in 30 consecutive human lesions.

**Results:**

The* in-vitro* experiments demonstrated that MLD and MLA after LSRA+HSRA were significantly larger (MLD: LSRA+HSRA=1.50 ±0.05 mm, HSRA= 1.43 ±0.05 mm, p=0.015; MLA: LSRA+HSRA= 1.90 ±0.17 mm^2^, HSRA= 1.71±0.11 mm^2^, and p= 0.037), requiring more crossing attempts (LSRA= 134 ±20 times, HSRA= 72 ±11 times, and p< 0.001). In human studies, there was no significance in reference vessel diameter and lesion length before the procedure between two groups. MLDs after LSRA+HSRA were significantly larger than those in HSRA (LSRA+HSRA= 1.22 ±0.16 mm, HSRA= 1.07 ±0.14 mm, and p= 0.0078), while MLAs after LSRA+HSRA tended to be larger (LSRA+HSRA= 1.79 ±0.51 mm^2^, HSRA= 1.55 ±0.47 mm^2^, and p= 0.19). There was no significance in the occurrence of in-hospital complication, including slow flow or no reflow, major dissection, and procedural myocardial infarction, between LSRA+HSRA and HSRA.

**Conclusions:**

LSRA can achieve larger lumen gain compared, whereas HSRA can pass calcified lesions easily. Combination of LSRA and HSRA is a safe and feasible strategy for severely calcified lesions in clinical practice.

## 1. Introduction

Rotational atherectomy (RA) is a method to ablate resistant or heavily calcified lesions mechanically. RA produces lumen enlargement by physical removal of plaque and reduction in plaque rigidity, enabling dilation [1]. However, universal adoption of RA technique has been hampered by lack of standardized protocols [2]. Traditionally, high-speed RA (HSRA) has been performed in the recommended range from 180,000 to 200,000 rpm. HSRA enables treatment of heavily calcified lesions, facilitating drug-eluting stent implantation and expansion [3, 4]. However, recent studies show that a safe range of RA speed is between 135,000 and 180,000 rpm. Lower platform speeds are reported to be associated with disadvantages, such as burr lodging and difficulty in passage of the burr to the distal lesion, while high platform speed increases platelet activation and thrombotic complications, such as slow flow and no reflow [5–7]. Although recent studies have led to a better understanding of optimal platform speed, little is known about the association between platform speed and acute lumen gain.

The objective of this study is to evaluate the effect of additional low-speed RA (LSRA) following conventional HSRA on acute lumen gain using sequential optical frequency domain imaging (OFDI) in* in-vitro* and human studies. To the best of our knowledge, this is the first report demonstrating the potential of this novel technique to treat severely calcified lesions with RA.

## 2. Methods

### 2.1. *In-Vitro* Studies

#### 2.1.1. Calcification Model

**Figure 1:**
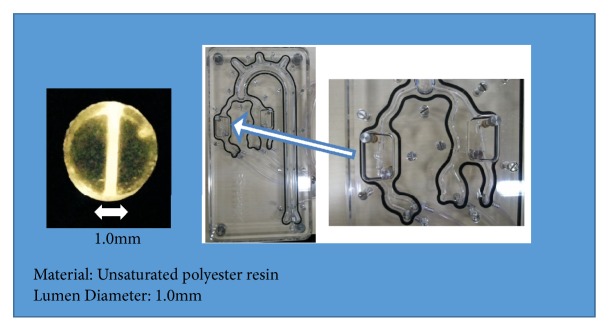
*Specifications of In-Vitro Calcified Model.* The artificial model was made of unsaturated polyester resins. A calcification model of coronary artery with 1.0 mm inner diameter was used in this study.

A calcification model of a coronary artery with 1.0 mm inner diameter was used. The artificial model was made of unsaturated polyester resins (Figure 1). A new model was installed into the model artery fixture for each test.

#### 2.1.2. Rotablator Systems

The Rotablator™ rotational atherectomy system (Boston Scientific Corporation, Natick, MA, USA) was used for this experiment. A RA catheter with a 1.5 mm burr and a Rotawire floppy guidewire (Boston Scientific Corporation, Natick, MA, USA) were connected to a console to power the device and to a water source to cool the system. The experimental set-up is shown in Figure 1. The air turbine was driven by compressed air from the air pressure regulator. Water at 25°C flowed through the introducer at 1-2 ml/sec to simulate blood flow.

#### 2.1.3. Optical Frequency Domain Imaging (OFDI)

Because of its high resolution (10 -15 *μ*m) could detect a clear inner border of the vessel lumen and quantify lumen area precisely. OFDI examination was performed using a FastView™ imaging catheter (Terumo Corporation, Tokyo, Japan). A bolus intracoronary injection of isosorbide dinitrate was administered prior to the OFDI examination. After manual calibration, the OFDI catheter was advanced more than 5 mm distally to the target lesion over a Rota floppy wire. Contrast was flushed through the guide catheter at a rate of 3 –4 ml/sec for approximately 3 –4 s using an autoinjector system. When a blood-free image was obtained, the OFDI core was pulled back over a longitudinal distance at a rate of 20 mm/sec.

#### 2.1.4. Test Procedures

A previous report showed that friction between the burr and plaque generates heat in the experimental model [4]. Since heat varies with technique from 2.6°C using intermittent ablation and permitting minimal decelerations (4,000-6,000 rpm), to 13.9°C using continuous ablation allowing excessive decelerations (14,000-18,000 rpm), intermittent ablation with a pecking motion was performed in i*n-vitro *and human studies. The number of pecking motion was examined between HRSA (190,000 rpm) and LSRA (110,000 rpm) using 1.5 mm RA burr. Then LSRA following HSRA (190,000 rpm followed by 110,000 rpm) was applied. HSRA, LSRA following HSRA, and LSRA were examined in quintuplicate. All HSRA procedures were performed until the decrease of rotational speed was lower than 1,000 rpm without generating friction during burr runs. In all 5 experiments with LSRA following HSRA, some friction was observed but passage of the 1.5 mm burr to the distal lesion at 110,000 rpm followed by 190,000 rpm was demonstrated. Maximum decrease in burr rotational speed after 110,000 rpm was 3,600 ±894 rpm. LSRA was applied until the decrease of rotational speed was lower than 1,000 rpm without friction occurring during burr runs. Before and after each ablation, OFDI was performed to evaluate MLD and MLA.

### 2.2. Human Studies

#### 2.2.1. Study Population

**Figure 2:**
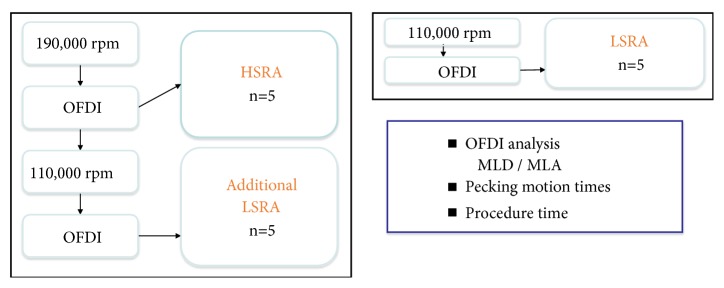
*Study Flowchart of In-Vitro Calcified Model.* OFDI= optical frequency domain imaging; HSRA= high-speed rotational atherectomy; LSRA= low-speed rotational atherectomy; MLD= minimum lumen diameter; MLA= minimum lumen area.

Figure 2 shows the study flow chart. We retrospectively enrolled 30 consecutive lesions in 29 patients who underwent RA with a 1.5 mm burr from June 2013 to January 2017 by using OFDI examination in a single center. The study was conducted in accordance with the provisions of the Declaration of Helsinki and approved by the Tokyo Medical and Dental University Hospital ethics committee, and all patients gave informed consent for their participation before the study.

#### 2.2.2. Percutaneous Coronary Intervention (PCI) Procedure

During the study period, a single operator (T. Ashikaga) in our institution performed all of the RA procedures. All patients received dual antiplatelet therapy of 100 mg aspirin and 75 mg clopidogrel before the procedure. Immediately before intervention, intra-arterial heparin was given to maintain an activated clotting time longer than 300 sec. RA was performed in conjunction with an intracoronary infusion of a “cocktail” containing verapamil, heparin, and nitroglycerin with burr runs within 10 sec in duration in order to avoid burr deceleration. Elective temporary right ventricular pacing was performed in a limited number of cases. The decision to perform RA-PCI was made at the operator's discretion, following prior visualization of a heavily calcified coronary lesion and failure to cross the lesion with an OFDI catheter. The lesion was crossed with a 0.014-inch guidewire, which was exchanged with a 0.009-inch Rotawire floppy guidewire using a microcatheter. Fifteen lesions were ablated at 190,000 rpm, defined as HSRA; and the remaining 15 lesions were ablated at 110,000 rpm following the ablation at 190,000 rpm, defined as LSRA following HSRA. The operator advanced and retreated the advancer knob 5 times within 10 sec to cause relatively slow back-and-forth pecking motion in each run of ablation. The runs of ablation were repeated as follows.

RA was performed avoiding decreases greater than 5000 rpm in the rotational burr speed. All HSRA procedures were performed until the decrease of rotational speed was lower than 1,000 rpm without friction occurring during burr runs. In the LSRA following HSRA group, 110,000 rpm ablation was applied. In all 15 lesions, some friction occurred but passage of the 1.5 mm burr to the distal lesion at 110,000 rpm could be accomplished. The maximum decrease in burr rotational speed after 110,000 rpm was 4,200 ±2,017 rpm. LSRA was applied until the decrease of rotational speed less than 1,000 rpm occurred without friction during burr runs. MLD and MLA were measured by OFDI immediately after RA.

#### 2.2.3. Statistical Analysis

Statistical analysis was performed using JMP statistics software version 9.02 (SAS Institute Inc.) All data are expressed as mean ±SD. Two-tailed paired Student's t-tests or nonparametric Wilcoxon tests were used to compare the significance of the differences.

## 3. Results

### 3.1. In-Vitro Study

**Figure 3:**
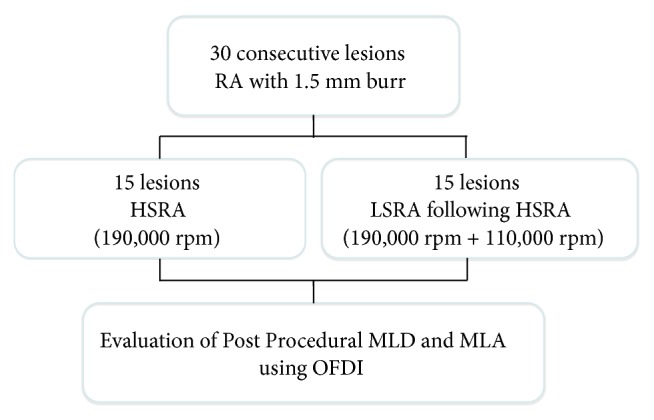
*Study Flowchart of Human Studies.* 30 lesions in patients who underwent rotational atherectomy (RA) with 1.5 mm burr from June 2013 to January 2017. 15 lesions were ablated at 190,000 rpm, defined as high-speed RA (HSRA), and 15 lesions were ablated at 190,000 rpm followed by 110,000 rpm, defined as additional low-speed RA (LSRA) following HSRA. Post-RA minimum lumen diameter (MLD) and minimum lumen (MLA) were measured immediately afterwards by optical frequency domain imaging (OFDI).

Table 1 demonstrates the comparison of the numbers of crossing attempts with pecking motion and total procedural time in the* in-vitro* experiments. More crossing attempts were required in the LSRA model to pass the lesion than in the HSRA model (LSRA= 134 ±20 times, HSRA= 72 ±11 times, and p< 0.001). The total procedure time was significantly longer in the HSRA model than in the LSRA model (LSRA= 55.2 ±14.2 sec, HSRA= 89.4 ±13.9 sec, and p< 0.010).  Figure 3 demonstrates the MLD and MLA just after RA in* in-vitro* experiments. MLD and MLA after HSRA immediately following LSRA (LSRA +HSRA) were significantly larger than those of HSRA alone in this* in-vitro* model (MLD: LSRA +HSRA= 1.50 ±0.05 mm, HSRA= 1.43 ±0.05 mm, and p= 0.015; MLA: LSRA +HSRA= 1.90 ±0.17 mm^2^, HSRA= 1.71 ±0.11 mm^2^, and p= 0.037).

### 3.2. Human Studies

#### 3.2.1. Patient Characteristics

**Figure 4:**
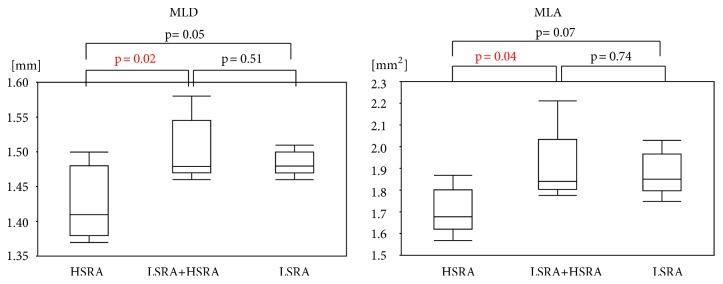
*In-vitro Experiment (MLD and MLA after Ablation with 1.5 mm Rotablator Burr).* Comparison of minimum lumen diameter (MLD) immediately after high-speed rotational atherectomy (HSRA), low-speed rotational atherectomy (LSRA) following HSRA.

The clinical study included 30 consecutive lesions in patients who underwent PCI using 1.5 mm RA burr for severely calcified lesions (Figure 4). There were no significant differences between the 2 groups in terms of baseline patient characteristics (Table 2).

#### 3.2.2. Procedural Results

More left anterior descending lesions were treated in both groups. Details of baseline quantitative coronary angiographic (QCA) analysis are listed in Table 3. Regarding reference vessel diameter (RVD), preprocedural MLD, and lesion length by QCA, there were also no significant differences between the 2 groups (Table 3). The number of pecking motion times in LSRA +HSRA group were significantly larger than that in HSRA only group (LSRA +HSRA= 48.8 ±29.6, HSRA= 33.2 ±12.3, and p= 0.05).

#### 3.2.3. OFDI Results

**Figure 5:**
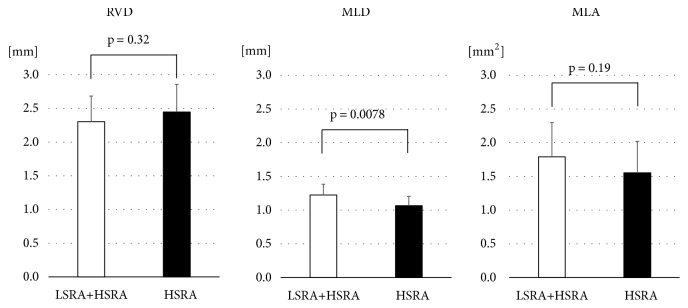
*Human Studies MLD and MLA after 1.5-mm Rotablator Burr.* Comparison of minimum lumen diameter (MLD) and minimum lumen is (MLA) after high-speed rotational atherectomy (HSRA), low-speed rotational atherectomy (LSRA) following HSRA. RVD = reference vessel diameter.

The postprocedural OFDI analyses for RVD, MLD, and MLA are shown in Figure 5. The postprocedural RVD was similar in both groups (LSRA +HSRA= 2.30 ±0.38 mm, HSRA= 2.45 ±0.41mm, and p= 0.32). The postprocedural MLD was significantly larger in LSRA +HSRA group compared to HSRA only group (LSRA +HSRA =1.22 ±0.16 mm, HSRA =1.07 ±0.14 mm, and p= 0.0078). The postprocedural MLA tended to be larger in LSRA +HSRA group than in HSRA group (LSRA +HSRA =1.79 ±0.51 mm^2^, HSRA= 1.55 ±0.47 mm^2^, and p= 0.19).

#### 3.2.4. In-Hospital Outcomes

There was no significant difference between two groups in the diameters of deployed stents (data not shown). The incidence of in-hospital complications is shown in Table 4. The incidence of slow flow or no reflow was not significantly different between the two groups. Although there was no significance, the occurrence of in-hospital complication, including slow flow or no reflow, major dissection, and procedural myocardial infarction, in LSRA +HSRA, were smaller than those in HSRA. No other major complications were observed in either group.

## 4. Discussion

Our present findings indicate that HSRA can pass the calcified lesions easily with this pecking motion technique. Additional LSRA can also pass the calcified lesion easily and achieve larger lumen gain compared with conventional HSRA alone. Previous studies reported that HSRA produces heat in the plaque and arterial endothelium, which may lead to activation of inflammatory factors as well as triggering the clotting cascade and platelet activation. Reisman et al. showed that platelet activation was decreased by lowering the rotational speed [5]. Williams et al. also showed that platelet activation is speed-dependent [6]. Considering these facts, LSRA was recommended to reduce platelet activation and thermal injury in clinical practice. A previous clinical study also reported that LSRA is superior to HSRA in terms of 1-year restenosis rate [7].

In contrast, HSRA is sometimes necessary to pass the RA burr easily for severely calcified lesions in daily practice because HSRA can facilitate longitudinal burr movement across calcific lesions by orthogonal displacement of friction. Our* in-vitro* study also demonstrated that HSRA can pass the calcified model easily. Since stent delivery and expansion are often difficult or even impossible without RA in heavily calcified lesions, to pass the calcified lesions to the distal lesion with RA is an important issue in clinical practice. Therefore, many interventionists prefer to perform HSRA in clinical practice in order to minimize time and complete accomplishment of RA.

We are very interested in LSRA because our experience has demonstrated that large lumens could be obtained using LSRA. While the reasons for this result are not yet entirely understood, rotor dynamics can provide a possible explanation. Rotating machinery such as those used in rotational atherectomy is subject to vibration because any rotor has some unbalance to a greater or lesser degree [8]. Our* in-vitro* and human studies demonstrated that LSRA following HSRA can produce higher acute lumen gain. We assumed that 110,000 rpm would produce more vibration amplitude than 190,000 rpm, which would lead to higher acute lumen gain.

HSRA at 140,000 -150,000 rpm is recommended in the current expert opinion and Sakakura et al. compared HSRA at 140,000 rpm with HSRA at 190,00 rpm and reported no reduction of incidence of slow flow or myocardial infarction in HSRA at 140,000 rpm [1, 9]. Even though HSRA at 140,000-150,000 rpm is safe enough and able to gain successful results, LSRA +HSRA strategy is able to produce higher acute lumen gain without burr sizing up. With respect to burr size, LSRA +HSRA strategy can reduce the incidence of procedural complication because HSRA with smaller burr size is reported to reduce procedural complications [10, 11].

The disadvantage of LSRA is that it takes more time than HSRA. Although Sakakura et al. showed that total run time was very similar between HSRA at 140,000 rpm and HSRA at 190,00 rpm, it can be assumed that total run time depends on the characteristics of coronary lesion [9]. Even so, it is certain that LSRA following HSRA can provide a larger lumen gain and save procedural time. For that reason, we planned and established the strategy to combine LSRA with HSRA. Even though the number of pecking motion times in LSRA +HSRA group was significantly larger than that in HSRA only group, we assume that the number of pecking motion times is smaller and the duration of total PCI procedure in LSRA +HSRA is shorter than those in LSRA only.

### 4.1. Study Limitations

Several limitations must be mentioned with regard to this study. This is a retrospective single-center study. The number of lesions was relatively small, and there was no randomization. However, in order to minimize variation in human factors, a single operator conducted the procedures. In addition, this study was limited to a 1.5 mm burr size. Some procedures also required other burr sizes, and some friction was generated, but passage of the other burr sizes to the distal lesion at a speed of 110,000 rpm could be accomplished in our experience. A prospective randomized trial is required.

## 5. Conclusions

This study suggests that LSRA following HSRA can achieve larger lumen enlargement compared with conventional HSRA. In order to reduce the repetition of pecking motion, it is better to start the ablation at high speed and then reduce the speed afterwards.

## Figures and Tables

**Table 1:** *In-vitro *experiment.

	HSRA (n= 5)	LSRA (n= 5)	P value
Pecking motion times	72.2 ±10.8	133.6 ±20.4	< 0.0001

Procedural time (sec)	55.2 ±14.2	89.4 ±13.9	< 0.01

Values are mean ±SD.

HSRA= high-speed rotational atherectomy and LSRA= low-speed rotational atherectomy.

**Table 2:** Baseline characteristics (n= 30 lesions).

	HSRA (n= 15)	LSRA+ HSRA (n= 15)	P value
Age (yrs)	73.0 ±9.6	73.0 ±8.9	0.76

Female	4	8	0.14

Transradial approach	11	9	0.44

Body mass index(kg/m2)	23.6±2.3	23.4±4.7	0.72

Hypertension (%)	14 (93)	13 (87)	0.54

Dyslipidemia, n (%)	10 (67)	10 (67)	1.00

Diabetes mellitus, n (%)	7 (47)	5 (33)	0.12

Prior MI, n (%)	3 (20)	4 (27)	0.46

CCS III/IV, n (%)	5 (33)	6 (40)	0.67

LV ejection fraction (%)	62.0±5.4	63.0±11.5	0.29

Multivessel disease, n (%)	12 (80)	9 (60)	0.23

Values are n (%) or mean ±SD.

HSRA= high-speed rotational atherectomy, LSRA +HSRA= low-speed rotational atherectomy following HSRA, LV= left ventricle, CCSIII/IV= Canadian Cardiovascular Society grading III/IV of angina pectoris, MI= myocardial infarction.

**Table 3:** Angiographic and procedural characteristics (QCA) (n= 30 lesions).

	HSRA (n= 15)	LSRA +HSRA (n= 15)	P value
Location			0.31
Left anterior descending	12 (80)	13 (87)	
Left circumflex	2 (13)	0 (0)	
Right coronary artery	1 (7)	2 (13)	

Guide catheter			0.07
6F	11 (73)	6 (40)	
7F	4 (27)	9 (60)	

Before procedure			
Pre-RVD (mm)	2.19 ±0.51	2.07 ±0.46	0.47
Lesion length (mm)	16.68 ±10.47	14.20 ±8.21	0.48
Pre-MLD (mm)	0.85 ±0.16	0.83 ±0.22	0.81

Pecking motion times*∗*			
190,000 rpm	33.2 ±12.3	36.9 ±31.5	0.58
110,000 rpm	-	11.9 ±4.3	-
total	33.2 ±12.3	48.8 ±29.6	0.05

Immediately after RA			
Post-MLD (mm)	1.24 ±0.29	1.34 ±0.33	0.38

Values are n (%) or mean ± SD.

HSRA= high-speed rotational atherectomy, LSRA +HSRA= low-speed rotational atherectomy following HSRA, MLD= minimal lumen diameter, QCA= quantitative coronary angiography, RA= rotational atherectomy, and RVD= reference vessel diameter.

*∗*Pecking motion times data were not available in 1 case in LSRA +HSRA group. They were excluded in the analysis of pecking motion times comparison between the groups.

**Table 4:** In-hospital outcomes.

	HSRA (n= 15)	LSRA+HSRA (n= 15)	P value
Slow flow or no reflow	4 (27)	2 (13)	0.36

Major dissection	1 (7)	0 (0)	0.31

Perforation	0 (0)	0 (0)	1.00

Side branch occlusion	0 (0)	0 (0)	1.00

Procedural MI	2 (13)	2 (13)	1.00

Values are n (%) or mean ±SD.

HSRA= high-speed rotational atherectomy, LSRA +HSRA= low-speed rotational atherectomy following HSRA, MI= myocardial infarction.

## Data Availability

The Excel data used to support the findings of this study are available from the corresponding author upon request.

## Abbreviations

HSRA: High-speed rotational atherectomy
LSRA: Low-speed rotational atherectomy
MLA: Minimum lumen area
MLD: Minimum lumen diameter
OFDI: Optimal frequency domain imaging
QCA: Quantitative coronary angiographic
RA: Rotational atherectomy
RVD: Reference vessel diameter.
